# Exosomal Circular Ribonucleic Acid–Microribonucleic Acid Expression Profile from Plasma in Alzheimer’s Disease Patients by Bioinformatics and Integrative Analysis

**DOI:** 10.5152/eurasianjmed.2023.23029

**Published:** 2023-10-01

**Authors:** Nail Besli, Bahar Sarikamis, Rabia Kalkan Cakmak, Ulkan Kilic

**Affiliations:** 1 Department of Medical Biology, University of Health and Sciences Institute of Health Sciences, İstanbul, Turkey; 2 Department of Medical Biology, University of Health Sciences Hamidiye Faculty of Medicine, İstanbul, Turkey

**Keywords:** Plasma, exosomal circRNAs–miRNAs, bioinformatics analysis, Alzheimer’s disease

## Abstract

**Objective:**

Alzheimer’s disease is a neurodegenerative sickness and increasing with age throughout the world. A substantial body of evidence suggests the role of exosomal noncoding ribonucleic acids in the development of Alzheimer’s disease, but the regulatory mechanisms mediated by these noncoding ribonucleic acids remain extensively unknown. Using plasma samples from Alzheimer’s disease patients, this study explored the exosomal circular ribonucleic acid–microribonucleic acid profiles.

**Materials and Methods:**

The ArrayExpress platform was used to convey data from 3 samples from each group (healthy, mild cognitive impairment, and Alzheimer’s disease). Using plasma exosomes, differentially expressed microribonucleic acids and differentially expressed circular ribonucleic acids were compared between the Alzheimer’s disease and mild cognitive impairment groups. Afterward, to define pathways, gene ontologies, and networks, differentially expressed microribonucleic acids and differentially expressed circular ribonucleic acids common to both mild cognitive impairment and Alzheimer’s disease groups were analyzed. Eventually, the selection of hub genes and protein–protein interaction network was analyzed.

**Results:**

A total of common 19 (7 upregulated and 12 downregulated) differentially expressed microribonucleic acids and 24 differentially expressed circular ribonucleic acids were recognized. A total of 4559 target genes were predicted for upregulated differentially expressed microribonucleic acids, while 6504 target genes were identified for downregulated differentially expressed microribonucleic acids, and most of the target genes involved in the phosphoinositide 3-kinases-Akt pathway and that were mostly regulated by hsa-mir-374a-3p, mir-196a-5p, let-205-5p, mir-185-3p, mir-374a-5p, mir-615-3p, let-7c-5p, mir-185-5p. Additionally, 9 hub genes (HSP90AA, ACTB, MAPK1, GSK3B, CCNE2, CDK6, AKT1, IGF1R, CCND1) were revealed as the genes considerably related to Alzheimer’s disease by a protein–protein interaction network using the cytohubba in Cytoscape software.

**Conclusion:**

Our findings provide a new perspective on how microribonucleic acids could connect with circular ribonucleic acids in the pathogenesis of Alzheimer’s disease.

## Introduction

In terms of dementia, Alzheimer’s disease (AD) is the most common form and is incurable with the prevalence rising as the population ages. An increasingly recognized global health problem, AD is a chronic disorder that causes amyloid beta plaques and neurofibrillary tangles of hyperphosphorylated tau to form.^1,2^ There is growing interest in identifying early screening biomarkers for diseases such as AD, where cognitive impairment tends to occur later in life than actual disease progression.^3^

**Main Points**

Our present in silico analysis has highlighted several miRNA-mRNA pathways and integration of miRNA-circRNA as possible causes of the pathology of Alzheimer’s disease.
19 DEmiRs and 24 circRNAs were found in the plasma exosome in both the AD and MCI groups compared with the healthy group.
To the best of our knowledge, mir-let-7c and mir-205 have not previously been associated with AD and they might be potentially novel biomarkers.
According to the statistically significant miRNA and circRNA network, most of the target genes were observed to be related to the PI3K-Akt pathway.

Known as vesicles and variable-membrane structures, exosomes are small vesicles that shuttle in the extracellular space, forming an existing intercellular connection.^4^ Due to the fact that exosomes contain an ingredient that changes depending on their origin and destination, they may serve as biomarkers for detecting early AD.^3^ Interestedly, the use of exosome-based biomarkers for the diagnosis of neurodegenerative disease is a current and rapidly developing field given that exosomes most often cross the blood–brain barrier.^3^ Thus, exosomes are well-considered to conduct a robust function as a drug delivery vehicle as well.

Recent advances in microarrays and next-generation sequencing (NGS) have led to the discovery of a few types of noncoding ribonucleic acids (ncRNAs) in several tissues and exosomes, such as microRNAs, circular RNAs (circRNAS), and long ncRNAs.^5^ Nevertheless, exosomes can protect plasma ribonucleic acids (RNAs) from degradation by preventing them from being degraded by ribonucleases (RNases), a large group of hydrolytic enzymes. Therefore, RNase activity can serve as a better indicator of pathological changes.^6^

MicroRNAs (miRNAs) are potent molecules that regulate genes by preventing translation or can be induced for degradation by identifying specific binding sites on messenger RNA (mRNA).^7^

A growing number of miRNAs within the nervous system, where they play a crucial role in processes such as neurogenesis, synaptic plasticity, differentiation of neurons, and neurite growth, contributes to understanding the hypothesis that miRNAs may play a role in neurodegenerative diseases, especially AD.^8,9^ Further, circRNAS are a special class of single-stranded ncRNAs that have conspicuous accumulation in the brain tissues and are associated with neurodegenerative disorders, like AD.^10^ A growing body of evidence indicates that circRNA is also implicated in many complex biological processes, including miRNA sponge function, competition for endogenous RNA, transcription regulation, and actually protein synthesis.^11,12^ While there is an abundance of new data about circRNAs, there has been little research into connecting circRNAs and miRNAs in order to improve the molecular regulatory mechanisms.

In the present work, we aimed to address the following concerns to support understanding of the initiating molecular mechanisms of AD and to contribute untested insights into likely therapeutic targets; to detect the miRNAs and circRNAs expressed exosomally in plasma, to determine whether RNAs transcribed in brain tissues were expressed in exosomes, to use the RNAs to form an integrative network to perform functional enrichment analysis as in Gene Ontology (GO) and Pathway.

## Materials and Methods

### Data Collection

**Figure 1.**
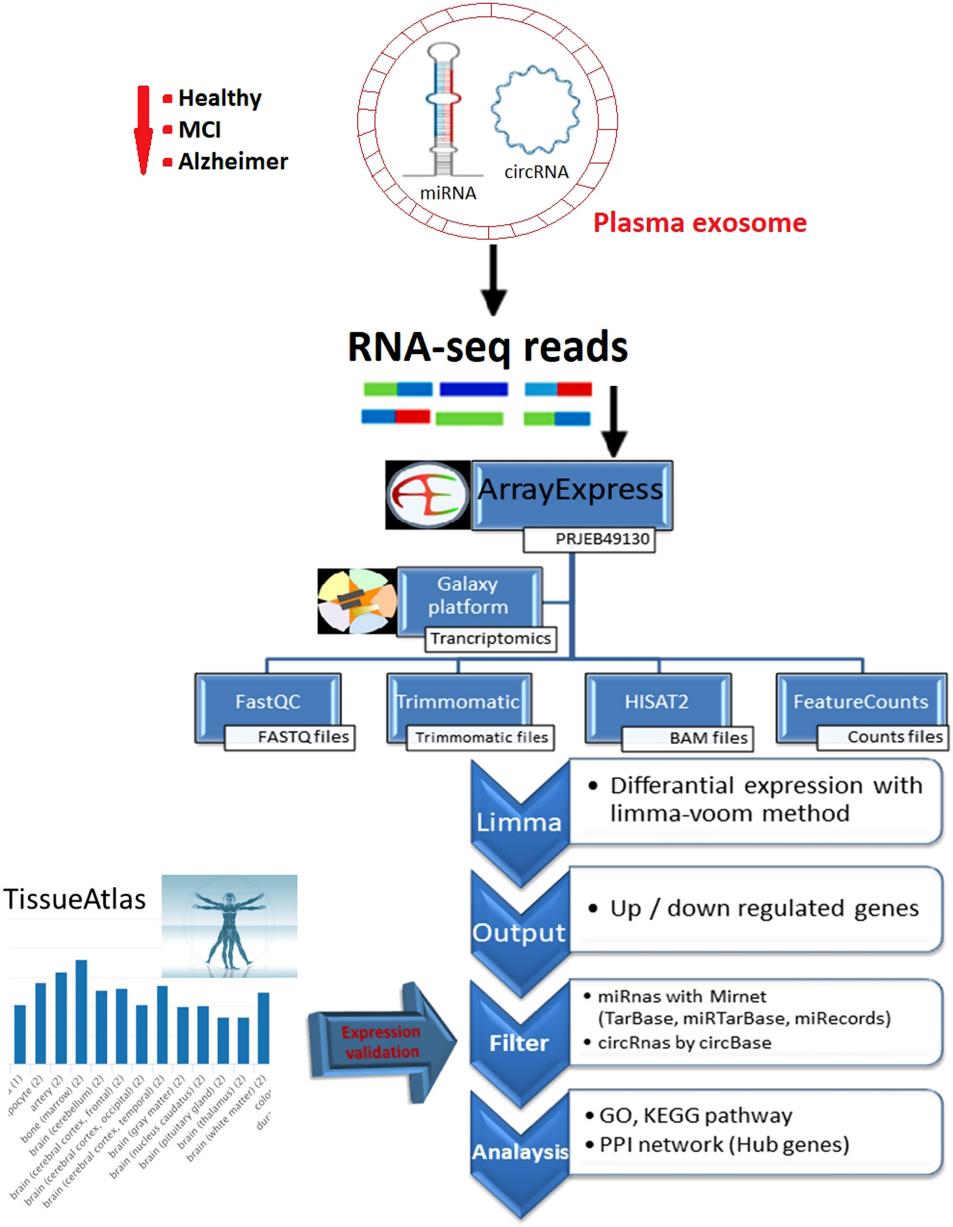
The pipeline of the present study consists of 3 main parts. The first step is differential expression gene analysis of sequencing exosomal RNA in AD patients. The second step is the integrative analysis of up- and downregulated genes. The third step is functional enrichment analysis of the gene sets. AD, Alzheimer’s disease; GO, Gene Ontology; KEGG, Kyoto Encyclopedia of Genes and Genomes; MCI, mild cognitive impairment; PPI, protein–protein interaction; RNA, ribonucleic acid.

A work schema of the paper is displayed in Figure 1. The data were received from the ArrayExpress (https://www.ebi.ac.uk/arrayexpress/experiments/E-MTAB-11222), which is stored for sequencing RNA data by operating the linkage to transmission to the galaxy online server.^13^ The E-MTAB-11222 dataset from the study of Xiaohuan et al (2022) consists of all 9 male samples, including 3 samples from each group (healthy, mild cognitive impairment (MCI), and AD respectively.^14^ In terms of the RNA-seq protocol, the library preparation was sequenced upon the platform of Illumina Hiseq 2500/2000, resulting in single-end reads of 50 bp. Based on the fastq data set, miRNA and circRNA differentially expressed genes were identified (differentially expressed microribonucleic acids (DEmiRs) and differentially expressed circular ribonucleic acids (DEcircRs)).

### Detection of Differentially Expressed Ribonucleic Acids

In Figure 1, the lists of DEmiRS and DEcircRs which are upregulated and downregulated genes compared to the healthy control group were created by using the following package programs FASTQC, Trimmomatic, HISAT2, FeatureCounts, and Limma in the Galaxy. The cutoff criteria were |log2(foldchange)| >0 and adjusted *P*-value < .05.

### Construction of the Networks and Module Analysis

A user-friendly bioinformatics tool that integrates data from 14 miRNA databases, miRNet (https://www.mirnet.ca), was utilized to predict the target genes of probable DEcircRs and DEmiRs.^15^ Two gene lists of up- and downregulated DEmiRs were individually tested in the miRNet. In order to monitor possible network integrations, DEmiRs and DEcircRs, however, were screened as multiple query types. The online server (https://molbiotools.com/listcompare.php) was used to sort the intersection of miRNet predictions of target genes. Thereafter, using the StringApp,^16^ the protein–protein interaction (PPI) network of target genes was formed,^17^, ^18^ hub genes in the network were identified by analysing the Maximal Clique Centrality (MCC) connectivity model with the Cytoscape 3.9.1. plug-in cytohubba.^19^

### Conformation Expression of Differentially Expressed Circular Ribonucleic Acid and Differentially Expressed Microribonucleic Acid Genes

The TissueAtlas^20^ network was used to confirm the expression of DEcircRs and DEmiRs take place in the brain. Target genes of these RNAs were extracted from the circBase database.^20^

### Pathway and Functional Enrichment Analyses

Functional annotation and pathway enrichment analysis for the target genes of DEmiRs and DEcircRs was conducted via DAVID^21^ (http://david.abcc.ncifcrf.gov) database. Gene Ontology^22^ and Kyoto Encyclopedia of Genes and Genomes (KEGG) terms with the *P*-value < .05 were accepted as significant.

## Results

### Constructing of Predicted Target Genes of Exosomal Differentially Expressed Microribonucleic Acids and Differentially Expressed Circular Ribonucleic Acids in Networks

**Figure 2. A-B.**
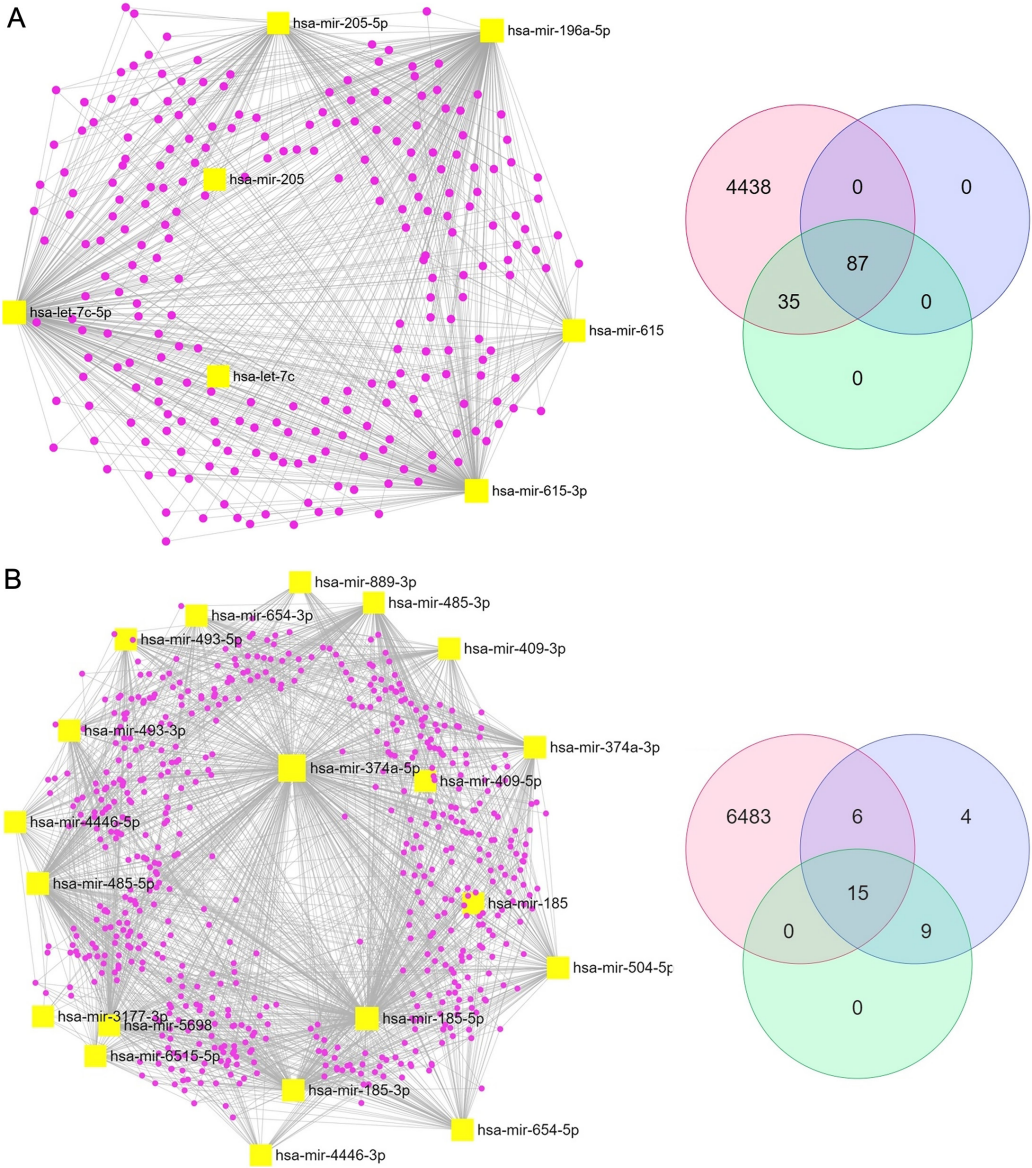
A Venn diagram showing DEmiRs and DEcircRs, and target genes of DEmiRs that appear in the networks for patients with MCI and AD, showing the intersection of potential target genes of upregulated miRNAs, target genes of upregulated miRNAs; 4559 (pink), neuronal circRNA genes (blue); 87, circRNA genes of upregulated miRNAs; 122 (green) (A), and target genes of downregulated miRNAs; 6504 (pink), neuronal circRNA genes (blue); 24, exosomal-upregulated circRNA genes in both AD and MCI group; 34 (green), showing the target genes of downregulated miRNAs (B). AD, Alzheimer’s disease; circRNA, circular ribonucleic acid; DEcircRs, differentially expressed circular ribonucleic acid; DEmiRs, differentially expressed microribonucleic acid; MCI, mild cognitive impairment; miRNA, microribonucleic acid;

In the present paper, plasma exosomes from 60-year-old men who have AD or MCI were compared with samples from 60-year-old men who are healthy for their miRNA expression profiles were analyzed. Table 1 shows that following a primary evaluation of the E-MTAB-11222 dataset, 7 upregulated and 12 downregulated DEmiRs were detected. Based on data from the miRNet platform, 4559 target genes were predicted for upregulated DEmiRs (Figure 2A) and 6504 target genes for downregulated DEmiRs (Figure 2B). Furthermore, a Venn diagram analysis of these genes in Figure 2B revealed that 21 out of 6504 target genes were exosomal upregulated circRNA genes, whereas 433 circRNAs were among 6504 target genes of upregulated miRNAs. In addition, as shown in Figure 2A, 122 of the 4559 miRNA targets are circRNAs.

As can be understood from Table 2, DEcircRs levels in the AD group were statistically more significant than the MCI group when compared to the healthy group. In the meantime, it was predicted by using circBase platform that 24 out of 34 common DEcircRs had neuronal origin.

### Functional Enrichment Analysis of the Predicted Target Genes of Up-/Downregulated Microribonucleic Acids

After the up- and downregulated miRNAs were submitted to miRNet, the degree cutoff was set to 2.0 for analysis of the 2 networks with better performance, resulting in 2 smaller networks of 625 genes and 250 genes.

Target genes of up-/downregulated miRNAs were subjected to functional enrichment analyses by using DAVID, GO category, and KEGG analysis and results are summarized in Tables 3 and 4. Furthermore, target genes of DEmiRNas related to AD were revealed by DISGENET database.

**Figure 3. A-B.**
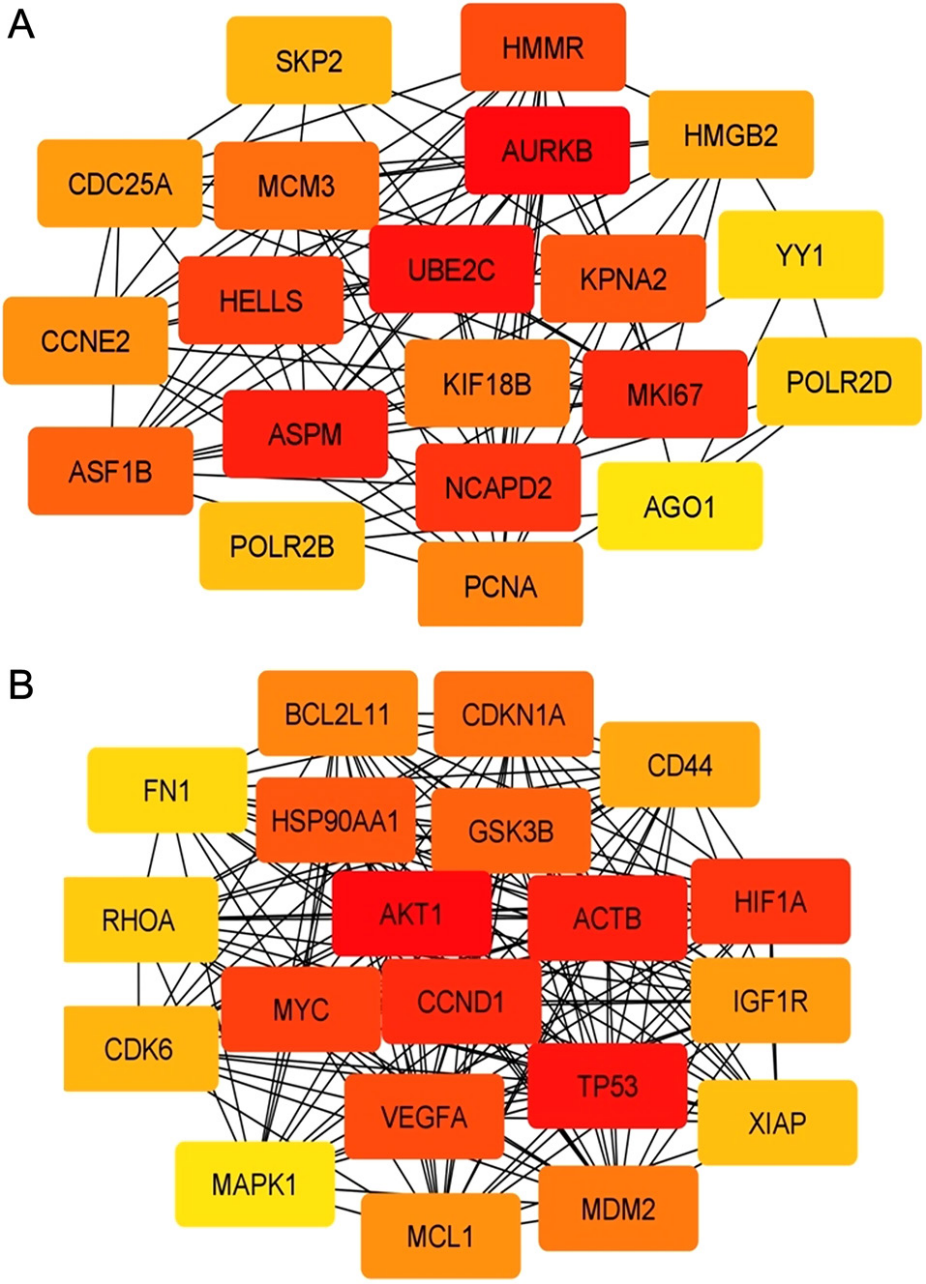
The illustration shows subnetworks acquired from the networks of target genes of upregulated and downregulated miRNAs, respectively, using the MCC model in the Cytoscape. Potential downregulated hub genes in a protein–protein interaction network (PPI) (A). Possible upregulated hub genes in a network of PPI. The grade of node color displays the degree of connectivity: orange color presents the intermediate degree, yellow color indicates the lowest degree, and red color represents the highest degree (B).

**Figure 4.**
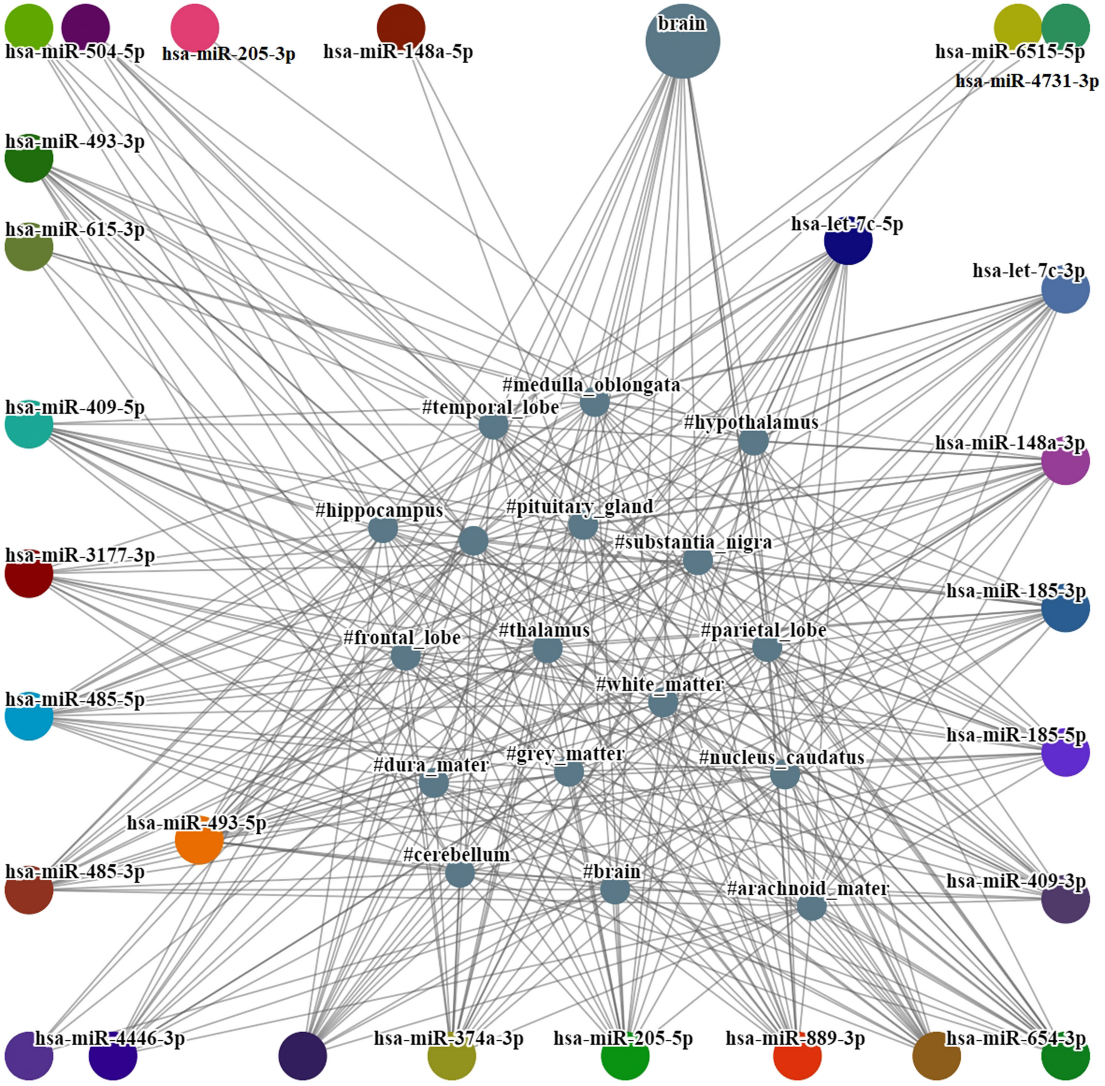
TissueAtlas shows statistically significant correlations between miRNAs upregulated or downregulated in exosomes. As can be seen that DEmiRs are colored nodes, and some of these mature miRNAs were derived from DEmiR precursors. RPMM (Reads Per Million Reads) and Min Expression ≥ 2. DEmiR, differentially expressed microribonucleic acid; miRNAs, microribonucleic acids.

As shown in Figure 3, the top 20 hub genes are divided into 2 subnetworks: an upregulated miRNA network identified in differential gene analysis with Cytoscape software that consists of 250 nodes and a downregulated miRNA network that consists of 625 nodes. Based on differential gene analysis by Cytoscape in Figure 3, the top 20 hub genes are divided into 2 subnetworks, that is, the STRING PPI network of 250 miRNA nodes and the STRING PPI network of 625 miRNA nodes. Validation of DEmiR expression in brain regions is shown in Figure 4. In the list of DEmiRs screening by TissuAtlas, some DEmiRs were mature miRNA precursors.

## Discussion

Multiple molecular factors contribute to the molecular complexity of Alzheimer’s pathophysiology. Further, since AD has been around as a health issue for many years, there is no reliable evidence of a cure. In spite of this, it may be predicted with the help of some molecular clues previous to reaching late AD stages, which would enable patients with AD to improve their quality of life. Therefore, by understanding the mechanism of AD through intercellular shuttled molecules, the complexity of molecular machinery can be enlightened.^3^ In order to identify miRNA–target interactions based on experimentally validated databases, our paper contributes to that issue by investigating plasma exosomal ncRNA expression profiles in AD patients.

**Figure 5.**
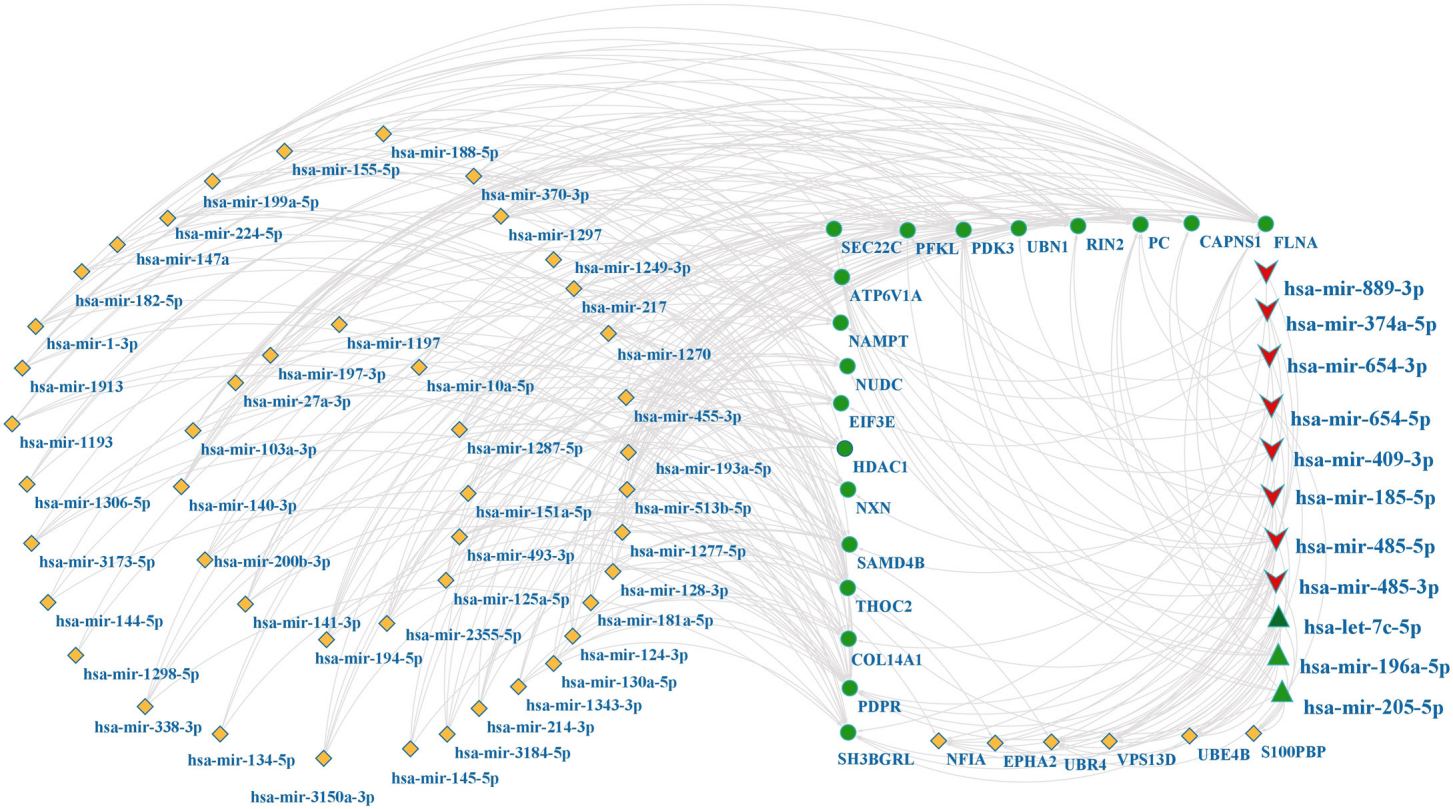
The miRNet tool illustrates potential interactions between the DEcircRs and DEmiRs, as a result of multiple interrogations of DEcircRs and DEmiRs, The red miRNAs are downregulated, while the green miRNAs are upregulated. Based on the miRTarbase v8.0 database, orange nodes indicate possible interaction genes (miRNAs and mRNAs) between DEcircRs and DEmiRs. DEcircRs, differentially expressed circular ribonucleic acids; DEmiRs, differentially expressed microribonucleic acids; miRNAs, microribonucleic acids.

In this study, we found commonly the 24 circRNAs and 19 DEmiRs in both the patients of MCI and AD samples in the plasma exosome compared to the healthy group via NGS data. In theory, deeming that secretion of exosomes from brain tissues to the blood, we determined that 15 upregulated circRNAs in Table 2 were of neuronal origin by scanning the circBase database, and these circRNAs were regulated by some downregulated miRNAs (see Figure 5). In addition to identifying the cell type expressing the upregulated circRNAs, circRNA genes were queried in the Single-cell Atlas of the Entorhinal Cortex in the Human Alzheimer’s Disease database^23^ and 26 of 34 circRNA genes were revealed in the data set (Supplementary Figure 1).

The precedence in AD physiology courses is to consider the misregulation and dysfunction of beta-amyloid and tau protein.^2,3^ However, there is not enough progress in understanding Alzheimer’s disease and it is unclear why AD pathology occurs. In the issue, 10 AD-related genes explained in Table 4 with an enrichment score of 1.69 were regulated by human miRNAs (mir-5698, mir-185, mir-504, mir-3177-3p, mir-374a, mir-493-3p, mir-409, mir-4446-5p, and mir-485) in the DEmiRs (see supplementary Figure 3). In the sub-network, the genes of GSK3B, IGF1R, and VEGFA are associated with phosphoinositide 3-kinases-Akt (PI3K-Akt) signaling pathway as well and regulated by mir-374a-5p, mir-504, and mir-5698.

CDK6, CCND1, CCNE2, MDM2, CDKN1A, and CCND2 are commonly regulated target genes of upregulated miRNAs in both p53 and PI3K-Akt signaling pathway, and mir-205-5p, mir-196a-5p, and let-7c-5p are downregulated the CCNE2 that is a hub gene. As is related to AD, the downregulated DEmiRs of let-7c-5p, mir-615-5p, mir-615-3p, and mir-196a-5p were responsible for negatively regulating apoptosis in MYC, MDM2, CCND2, PRLR, CDKN1A, and IGF1R genes in PI3K-Akt. It unveiled some hub genes among the network of target genes of downregulated miRNA such as MYC, IGF1R, CDKN1A, and MDM2 genes. As a result of this unexpected finding, it shows that the same genes might be found within both networks of DEmiRs that are up- and downregulated, as former scientific reports have indicated that several pathologic conditions have been associated with gene regulation by multiple miRNAs.^24^ The regulation of cell division and cell cycle are processed by CDK6, CCNE2, and CCND1 in the hub genes related to the PI3K-Akt pathway. Additionally, emerging evidence indicates that circRNAs reportedly regulate transcription as one of their complicated roles in biological functions.^11^ Among the targets of upregulated miRNAs, 17 genes are negatively regulated by the RNA polymerase II promoter, whereas 12 genes from circRNAs are positively regulated by the DNA template. On the other hand, hsa-let-7c-5p, hsa-mir-205-5p, hsa-mir-196a-5p, hsa-mir-615-3p regulated XBP,^25^ PSMD8, TUBB, CSNK2A1 and APP,^26^ WIPI1 and NRAS,^27^ DVL3,^28^ CALM1^29^ in the key pathway of AD (Supplementary Figure 4). There was a statistically significant increase in the mature forms of let-7c, mir-205, mir-196a-1, and mir-615 in exosomes of the AD group compared to the healthy group (see Table 1).

It is generally established that miRNA expression and the expression of the target genes are inversely linked. Based on this understanding, the KEGG pathway enrichment analysis revealed that the downregulated targets were enhanced in pathways related to PI3K-Akt, p53, cellular senescence, insulin signaling, and mTOR signaling, whereas the downregulated targets were predominantly enriched in PI3K-Akt and p53 signaling pathways. mir-185-5p and mir-374a-5p, which are both implicated in apoptosis and cellular senescence, had the most target genes for the 14 downregulated DEmiRs, each having 8 (Supplementary Figure 5). mir-485-5p from downregulated DEmiRs is regulated TP53 in the cellular senescence pathway (see Figure 5) and notably some neuronal circRNAs in Table 2. As part of the ER stress response to the apoptosis pathway, BCL2L11 and BBC3 genes, as well as the BCL2L11 gene related to beta-amyloid, and the AKT1 gene that negatively regulates autophagy, which is the highest degree hub genes in the MCC model, were commonly downregulated by mir-185-5p and mir-185-3p, whereas mir-5698 was responsible for tau protein binding hub genes for MAPK1 and ACTB.

More crucially, despite mir-5698 being elevated in the MCI group, statistically speaking, we only saw a downregulation in the AD group. Interestingly, based on these circRNAs, a striking association between hub gene TP53 and UBN1 was found inside the cellular senescence pathway, as UBN1 plays a significant role in aging by producing age-related heterochromatin foci (SAHF), which shut down genes that promote proliferation.^30^ Besides, the DEcircR in plasma exosomes with the most statistical significance seems to be UBN1, despite being predominant in the nucleoplasm (Adjusted *P*-value = 1.35E-17).

There is a common regulation of insulin signaling, mTOR and PI3K-Akt pathways by mir-409-3p, mir-185-5p, and mir-185-3p in the hub genes of IGF1R, GSK3B, and AKT1. Recent studies have disclosed that plasma samples from AD patients are reduced in hsa-miR-185-5p, which has been shown to affect APP dysregulation^31^ and neurofibrillary pathology.^32^ As noted earlier, IGF1R and GSK3B in the mTOR pathway mediate the cellular response to beta-amyloid, and AKT1 and GSK3B are particularly activated in 3 pathways to regulate neuron death, among the various AD kinds that are associated to 10 genes. Similarly, THBS1, CREB3L2, BCL2L11, and HSP90B1, all take a part in ER stress by being involved in the PI3K-Akt signaling pathway, while BCL2L11, GSK3B, and IGF1R play a role in beta-amyloid response, and HSP90AB1, GSK3B, and HSP90AA1 positively regulate the kinase activity of tau protein. Overall, Supplementary Figure 5 illustrates potential targets of DEmiRs that are downregulated that are linked to the pathways shown in Tables 3 and 4.

In brief, the study examined exosomal circRNA and miRNA expression patterns among plasma samples correlated to AD using functional enrichment analysis. Accordingly, it was found that mir-615-3p, mir-374a-3p, let-205-5p, mir-196a-5p, mir-185-3p, let-7c-5p, mir-185-5p, mir-374a-5p, and mir-615-3p mostly regulate target genes associated with the PI3K-Akt pathway as indicated by the statistically significant miRNA and circRNA network. More importantly, analysis of 66 blood samples with AD in the study for hsa-mir-615-3p has been reported as potential biomarkers for early detection of AD.^33^ hsa-mir-196 and mir-185,^34^ and mir-374a^35^ were detected in the plasma exosomes in this investigation and were consistent with those of earlier studies with AD. mir-205 and mir-let-7c, which have not previously been linked to AD to the best of our knowledge, may represent a potential biomarker candidate. Among 19 DEmiRS found in our study; hsa-mir-615-3p,^33^ hsa-let-7c,^36^ hsa-mir-4371,^37^ hsa-mir-654,^38^ hsa-mir-504,^39^ hsa-mir-409,^39^ hsa-mir-485,^40^ hsa-mir-4446,^41^ hsa-mir-3177,^42^ and hsa-mir-185^32^ have been associated with AD before. Since all of these miRNAs are candidate biomarkers, none of them are clinically approved yet. Scientifically influencing the results of the study is, however, limited in some ways. In the first place, the reader should first note that the study relied on bioinformatic prediction tools to analyze DEmiRs and circRNAs; therefore, further experimental investigation is needed to validate the results. A second issue is that the databases did not contain adequate data in order to integrate all DEmiRs and circRNAs associated with AD. A third reason was that 9 tissue samples were used exclusively for the NGS, which did not provide a sufficient sample size.

For future studies, a case–control study can be performed by matching AD patients and their healthy elderly partners with a sufficient number of patients. The level of miRNA and circRNA in blood exosomes could be validated by NGS, while expression levels of miRNA target genes might be determined by appropriate molecular biology methods.

## Conclusion

In this article, from the plasma of AD patients, we conducted an exosomal differential expression gene (DEG) study followed by bioinformatic and integrative analysis. Our present *in silico* findings can identify a variety of possible miRNA–mRNA routes and miRNA–circRNA integrations that affect the pathophysiology of AD. Our outputs based on consequences are intended to broaden the scope of our knowledge of AD pathogenesis.

## Figures and Tables

**Table 1.** Common DEmiRs in the 2 Groups MCI and AD Compared to the Healthy Group

	log_2_FC	Adjusted *P* Value
Upregulated DEmiRs		
hsa-let-7c	0.99	2.61E-02
hsa-mir-615	6.39	1.89E-03
hsa-mir-205	5.48	1.42E-02
hsa-mir-4731	2.70	4.29E-03
hsa-mir-196a-1	2.46	1.89E-03
hsa-mir-6812	2.46	1.89E-03
hsa-mir-148a	0.78	4.29E-03
Downregulated DEmiRs		
hsa-mir-889	-4.87	1.28E-02
hsa-mir-654	-3.78	1.46E-04
hsa-mir-6515	-5.33	1.52E-03
hsa-mir-5698	-4.57	8.48E-03
hsa-mir-504	-4.50	4.51E-02
hsa-mir-493	-3.85	3.53E-02
hsa-mir-485	-2.41	3.00E-04
hsa-mir-4446	-2.27	1.28E-02
hsa-mir-409	-2.95	1.60E-09
hsa-mir-374a	-4.90	1.89E-03
hsa-mir-3177	-4.77	3.64E-02
hsa-mir-185	-1.97	5.97E-03

The statistical values belong to DEmiRs in AD group. Adjusted *P* value *P* < .05.(DEmiR; differentially expressed microribonucleic acids, FC; Fold Change, hsa; Homo sapiens, mir; micro RNA, let;let-7 family of microRNAs)

**Table 2.** List of Common Neuronal DEcircRs

Gene Symbol	MCI Group		AD Group		DEmiRs
	Adjusted *P Value*	log_2_FC	Adjusted *P Value*	log_2_FC	
**FLNA**	7.74E-03	1.63	1.10E-04	6.18	hsa-mir-196a-5p
**ATP6V1A**	4.57E-02	0.74	1.33E-02	2.82	hsa-mir-485-3p/5p hsa-mir-654-5p hsa-mir-409-3p
**CAPNS1**	4.57E-02	3.48	2.34E-04	6.24	hsa-mir-185-5p
**NAMPT**	4.57E-02	1.75	2.73E-03	5.56	hsa-mir-145-5p
**UBN1**	4.57E-02	0.97	1.35E-17	8.12	hsa-mir-485-5p
**COL14A1**	4.57E-02	0.74	3.54E-02	2.87	hsa-mir-654-5p
**SAMD4B**	4.57E-02	2.56	1.90E-03	2.46	hsa-mir-185-5p
**SEC22C**	4.57E-02	0.74	1.90E-03	2.46	hsa-mir-185-3p, hsa-mir-485-5p
**NXN**	4.57E-02	0.74	4.29E-03	2.70	hsa-mir-409-3p
**PFKL**	1.66E-02	1.49	1.90E-03	2.46	hsa-mir-654-3p hsa-mir-654-5p
**PDPR**	4.57E-02	2.62	1.90E-03	2.46	hsa-mir-185-5p
**RIN2**	4.57E-02	0.74	1.90E-03	2.46	hsa-mir-485-5p hsa-mir-485-3p
**RGS22**	4.57E-02	0.74	1.90E-03	2.46	
**CGGBP1**	4.57E-02	0.74	1.82E-04	6.10	
**EXPH5**	4.57E-02	0.74	1.90E-03	2.46	
EIF3E	4.57E-02	0.744	1.33E-02	2.82	
ADAMTS13	4.57E-02	0.74	1.90E-03	2.46	
THOC2	4.57E-02	0.744	1.90E-03	2.46	
HDAC1	4.57E-02	0.74	4.29E-03	2.71	
PC	1.67E-02	1.49	3.54E-02	2.87	
PDK3	4.57E-02	0.744	1.90E-03	5.65	
SH3BGRL	4.57E-02	0.744	4.29E-03	2.71	
NUDC	4.57E-02	0.74	1.33E-02	2.82	
CNTN4	4.57E-02	0.74	4.29E-03	2.70	

Upregulated circRNas are shown in bold font. Adjusted *P*value < .05 accepted as significant.

AD, Alzheimer’s disease; DEcircRs, differentially expressed circRNAs; DEmiRs, differentially expressed miRNAs; MCI, mild cognitive impairment; FC,fold change.

**Table 3.** The Target Genes for DEmiRs in Analysis of Functional Enrichment

GO	Term	Gene Count	*P*	Benjamini
BP	Neuron migration	11	2.00E-03	1.10E-01
BP	Response to ER stress	10	7.60E-04	7.00E-02
BP	Negative regulation of neuron apoptotic process	12	6.40E-03	1.90E-01
BP	Positive regulation of neuron apoptotic process	6	3.30E-02	4.70E-01
BP	Cellular response to beta-amyloid	7	2.60E-03	1.20E-01
BP	Central nervous system neuron axonogenesis	4	8.50E-04	7.00E-02
BP	Negative regulation of autophagy	7	4,00E-03	1.40E-01
MF	Tau protein binding	8	4.30E-04	1.80E-02
MF	Calmodulin binding	16	1.90E-03	4.70E-02
MF	Transcription factor activity, sequence-specific DNA binding	43	8.5E-8	1.4E-5
MF	Transcriptional activator activity, RNA polymerase II transcription regulatory region sequence-specific binding	36	1.7E-6	1.7E-4
MF	RNA polymerase II transcription factor activity, sequence-specific DNA binding	63	2.8E-4	1.3E-2
CC	Extracellular exosome	106	2.50E-07	1.50E-05
CC	Endoplasmic reticulum	45	2.30E-02	2.20E-01
CC	Cytoplasm	251	6.10E-18	1.20E-15
CC	Nucleoplasm	211	1.60E-22	4.70E-20
CC	Nucleus	283	1.30E-23	7.70E-21
BP	Cell division	19	7.40E-07	4.70E-04
BP	Negative regulation of apoptotic process	24	1.00E+01	5.90E-08
BP	Negative regulation of transcription from RNA polymerase II promoter	35	1.46E+01	9.50E-09
BP	Regulation of cell cycle	16	5.60E-06	2.60E-03
BP	Positive regulation of transcription, DNA-templated	23	1.40E-05	5,50E-03
MF	Protein kinase binding	22	1.30E-06	1.30E-04
MF	DNA binding	40	1.90E-07	2.40E-05
MF	SMAD binding	10	1.20E-08	2.10E-06
MF	Protein binding	202	1.50E-11	3.80E-09
MF	RNA binding	52	9.40E-12	3.80E-09
CC	Cytoplasm	103	1.10E-08	9.00E-07
CC	Cytosol	104	5.80E-09	5.90E-07
CC	Membrane	65	5.20E-10	7.00E-08
CC	Nucleoplasm	110	3.80E-22	7.70E-20
CC	Nucleus	142	7.10E-24	2.90E-21

The opted-enriched GO terms associated with AD are listed.

BP, biological process; CC, cellular component; DEmiRs, differentially expressed miRNAs; GO, Gene Ontology; MF, molecular function.

**Table 4.** Funtional Enrichment Analysis of the Target Genes of DEmiRs

Target genes of downregulated miRNAs					
Category	Term		Gene Count	*P*	Benjamini
KEGG pathway	Cellular senescence		18	5.50E-05	1.50E-03
KEGG pathway	PI3K-Akt signaling pathway		29	9.80E-05	1.80E-03
KEGG pathway	Apoptosis		12	1.10E-02	5.80E-02
KEGG pathway	Insulin signaling pathway		12	1.20E-02	5.80E-02
KEGG pathway	mTOR signaling pathway		13	1.20E-02	5.80E-02
Target genes of upregulated miRNAs					
KEGG pathway	PI3K-Akt signaling pathway		14	4.20E-03	6.20E-02
KEGG pathway	p53 signaling pathway		7	1.00E-03	2.80E-02
Target genes of up/downregulated miRNAs enrichment score: 1.69					
DISGENET	Alzheimer’s disease, Late onset	ADAMTS1, CALM1, DPYSL2, ENO1,GSK3B, IGF1R,PRNP,SLC30A6, SOD2,VEGFA	10	2.00E-02	4.00E-01
DISGENET	Alzheimer’s disease, Early onset		10	2.00E-02	4.00E-01
DISGENET	Alzheimer’s disease, Focal onset		10	2.00E-02	4.00E-01
DISGENET	Familial Alzheimer’s disease (FAD)		10	2.10E-02	4.00E-01
DISGENET	Alzheimer’s disease		10	2.30E-02	4.00E-01

The selected enriched GO terms related to AD are listed.

KEGG; Kyoto Encyclopedia of Genes and Genomes, DEmiRs, differentially expressed miRNAs; DISGENET; Disease Gene Net; miRNA, microribonucleic acid.
